# Unveiling Digital Transformation: A Catalyst for Enhancing Food Security and Achieving Sustainable Development Goals at the European Union Level

**DOI:** 10.3390/foods13081226

**Published:** 2024-04-17

**Authors:** Anca Antoaneta Vărzaru

**Affiliations:** Department of Economics, Accounting and International Business, University of Craiova, 200585 Craiova, Romania; anca.varzaru@edu.ucv.ro; Tel.: +40-7-7392-1189

**Keywords:** digital transformation, food, SDGs, DESI, food security, European Union

## Abstract

The digital revolution is reshaping various aspects of society, including having a profound impact on food security and the advancement of Sustainable Development Goals (SDGs). This study investigates the relationship between digital transformation, quantified through the components of the Digital Economy and Society Index (DESI), and SDGs related to food (SDG1, SDG2, SDG3, and SDG10), along with the overall SDG Index score. The data used for investigation are sourced from reports issued by the European Commission concerning DESI, as well as the SDG reports for the period from 2017 to 2022. The paper elucidates how different components of digitalization, such as connectivity, digital skills, internet usage, and digital public services, influence the attainment of food security objectives and broader sustainable development targets using structural equation modeling and cluster analysis. The findings underscore the pivotal role of digital technologies in enhancing poverty alleviation, health and well-being, and, in particular, mitigating inequality. This study contributes to understanding the complex relationship between digital transformation and food security, offering insights for policymakers, practitioners, and stakeholders aiming to leverage technology for advancing SDGs and fostering a more equitable and sustainable future.

## 1. Introduction

In the current context of uncertainties and rapid changes at the global level, a resilient food system is essential for ensuring food security and promoting sustainable development [1]. Governments must take proactive action to identify and address risks within the food system [2]. Building a resilient food system involves not only identifying and eliminating weaknesses but also creating mechanisms and structures that allow for rapid adaptation and recovery when facing uncertainty and changes [3,4]. Therefore, the concept of resilience provides a valuable approach to addressing the complexity and fragility of the food system and promoting a more robust and sustainable approach to food security [5,6,7].

Climate change, demographics, and pandemics have intensified pressure on global food systems. Recent data underscore the critical imperative to double food production by 2050 to meet the escalating demands of the worldwide population [8]. Concurrently, approximately one-third of all food produced globally, amounting to 930 million tons, is lost or wasted throughout the agri-food supply chain, resulting in approximately 800 million people experiencing hunger [9]. These challenges underscore the urgent need to address issues pertaining to food security, reduce food loss and waste, and sustainably manage natural resources within the agri-food sector. Furthermore, it is essential to recognize the significant environmental impacts associated with food production and consumption. Highlighting the importance of the twin transition—both social and digital—towards sustainable development can mitigate these environmental impacts [8]. These challenges entail the development of resilient and sustainable food systems that can cope with increased food demand and reduce environmental impact. This development involves innovations in food processing, adapting to climate change, and promoting more sustainable agricultural practices [10,11].

The use of digital technologies and innovation in the agri-food sector represents a crucial opportunity for addressing significant challenges related to food security, environmental sustainability, and reducing inequality [12,13]. Integrating digital solutions and adopting more efficient agricultural and food practices contribute to achieving global sustainable development goals and improving the quality of health and life [14]. This digitization can provide innovative solutions to challenges faced by the agri-food sector, such as climate change, natural resource management, and increasing food demand. Digitization in the agri-food sector presents innovative technology such as artificial intelligence, remote sensors, agriculture precision, and intelligent farming while also integrating social media for better communication and blockchain technology for reliable data collection and enhanced transparency.

Artificial intelligence enables farmers to analyze vast amounts of data to make data-driven decisions in real time [1]. Remote sensors provide continuous monitoring of crops, soil moisture levels, and environmental conditions, allowing for timely interventions and precise resource allocation. Agriculture precision technologies, such as GPS-guided machinery and variable rate application systems, enable farmers to optimize inputs, minimizing waste and maximizing productivity [6]. Smart farming integrates these technologies into comprehensive management systems, fostering sustainable practices and enhancing the resilience of agricultural production [8]. Social media platforms play a vital role in fostering communication and knowledge exchange among farmers, producers, distributors, and consumers, facilitating discussions on sustainable agriculture [15]. Social media also serves as a powerful marketing tool, enabling more comprehensive outreach and building consumer trust. Likewise, the adoption of blockchain technology ensures reliable data collection, enhances transparency, and strengthens security measures in the agri-food supply chain, bolstering consumer confidence and addressing concerns regarding data protection [16].

Adopting digital technologies creates more resilient, sustainable, and future-oriented food systems, thereby contributing to the achieving of the UN’s Sustainable Development Goals (SDGs) [17]. These technologies can improve decision-making processes and contribute to the development and implementation of more effective policies in all areas of SDG action. The use of these technologies can also pose challenges, such as data protection and information security, as well as equitable access to digital technologies, which need to be addressed to maximize the benefits of these tools in promoting sustainable development [13,14].

This paper aims to investigate the impact of digital transformation on food security and advancing SDGs in the European Union, focusing on the relationship between the Digital Economy and Society Index (DESI) and food-related SDGs. It addresses a gap in the academic literature by providing a detailed analysis of how different components of the digital economy, such as connectivity, digital skills, internet usage, and digital public services, influence the achievement of food security and SDGs. The paper stands out for its innovative approach in providing fresh perspectives on the critical role of digital technologies in poverty alleviation, health enhancement, and inequality reduction. These insights contribute to a deeper understanding of the multidimensional relationship between digital transformation and food security, laying the groundwork for more informed policymaking and strategic interventions.

The study posits two fundamental research questions that form the basis of its two hypotheses. First, it explores whether digital transformation significantly contributes to the achievement of food-related SDGs across European Union countries. Secondly, it investigates the possibility of categorizing European countries into distinct clusters based on their levels of digital economy development and their progress towards SDGs related to food security. These research questions guide the examination of the complex relationship between digital transformation, SDGs, and food security within the European context. Both research questions align with the European Union’s active efforts to address challenges within the agri-food system and its focus on research and innovation policy to promote sustainable, healthy, climate-resilient, and inclusive food systems [18].

This study’s importance lies in its comprehensive exploration of the pivotal role of digital transformation in shaping food security outcomes, its novel insights into leveraging digital technologies for achieving SDGs, and its contribution to advancing understanding in the field of sustainable development and digital transformation at the EU level.

## 2. Literature Review

### 2.1. The Implications of Digital Transformation on the Food System

Efficient risk management within the food system involves not only reacting to immediate shocks but also developing and implementing strategies for long-term adaptation and risk minimization. This action may include investments in research and technology to improve crop resilience to climate change or the development of food storage and distribution systems that are more robust against disruptions [1]. A resilient food system must be inclusive and equitable, ensuring access to quality and sustainable food for all members of society. This perspective entails considering the needs and vulnerabilities of different social groups, as well as promoting sustainable and responsible agricultural and food practices. By adopting integrated policies and strategies, governments can contribute to building a more robust and sustainable food system capable of addressing future challenges and uncertainties [6].

The issues highlighted by Oliver et al. [19] underscore the vulnerability of global and European food systems, as well as the urgent need to strengthen their resilience [20]. Natural crises and factors of economic and social instability can severely disrupt the functioning of the food system [21]. Food system resilience highlights the importance of assessing and understanding all factors that can influence the system’s capacity to adapt to and cope with future challenges [6,22,23]. Digital technologies used in agriculture, such as crop monitoring systems, data analysis, and agricultural e-commerce platforms, can contribute to more efficient resource management and increased resilience to climate change and other risks [12,24,25,26].

The issue of vulnerability and resilience in food systems is increasingly significant and timely [27], particularly concerning natural disasters exacerbated by climate change and social conflicts. For example, the ongoing Russia–Ukraine conflict has profound implications for global trade and food security, highlighting the interconnectedness of geopolitical tensions with agricultural production and distribution networks. In addressing these challenges, innovative methodologies and strategies play a crucial role in enhancing the resilience of agri-food systems. Research papers [28,29] provide valuable insights into approaches that can bolster the ability of food systems to withstand and recover from shocks.

Agriculture 4.0 is an emerging paradigm that promises to revolutionize the way operations are managed and conducted in agriculture and the food industry [30]. Adopting advanced technologies and digital transformation processes can increase efficiency, sustainability, and innovation in these critical sectors of the economy. However, it is essential to address the challenges and obstacles associated with digital transformation carefully. The identification and proper implementation of appropriate digital technologies are crucial for the success and adaptability of the industry to future changes [31].

The transition to Industry 4.0 and Agriculture 4.0 represents a significant change in how operations are managed and conducted in various sectors of the economy [32]. Advanced technologies such as artificial intelligence, the internet of things, cloud computing, and Big Data have a profound impact on how goods and services are designed, produced, and distributed. Regarding the food industry, this transformation opens up new opportunities for improving efficiency, quality, and sustainability in the food chain [33,34]. Companies in the food industry can optimize their production processes and better respond to consumer demand by implementing these technologies, contributing to increased competitiveness and the promotion of healthy and responsible eating [35]. This evolution brings significant benefits to emerging economies, contributing to their economic growth and integration into the knowledge and technology-based global economy [36]. Therefore, it is essential to continue investing in the development and implementation of these technologies [32].

Digital transformation represents an essential stage in the technological evolution of the industry, agriculture, and services, bringing concepts such as advanced automation, the internet of things (IoT), data analysis, agriculture precision, and smart farming. In the food processing sector, these technologies can improve the efficiency, quality, and safety of food products, contributing to the modernization of the entire food production and distribution chain [37]. Digital transformation represents a revolutionary paradigm in manufacturing and agriculture, bringing forth a series of innovations and advanced technologies that entirely transform the way industrial operations are managed and executed [38,39]. Implementing smart sensors and IoT technologies in the food production process can enable real-time monitoring of critical parameters and optimize operations to reduce the risks of food contamination or deterioration [39,40,41,42,43]. Simultaneously, employing artificial intelligence can contribute to improving quality control processes as well as optimizing production planning and the supply chain [44]. On the other hand, Big Data provides a rich source of information and insights for decision-making, helping companies identify hidden patterns and trends in their data [45]. Overall, the adoption of technologies and concepts associated with Industry 4.0 in the food industry promises significant benefits, such as enhancing operational efficiency, improving product quality, and reducing losses and food waste [37,46].

The food industry includes several stakeholders, from farmers to food production and processing companies [32]. Digital transformation is an essential tool for addressing the challenges and opportunities in the food and agricultural industry [47,48]. This tool not only contributes to meeting the increasing demand for food but can also promote more ecological and responsible agriculture that fulfills the needs and values of society [31,49,50].

Understanding the relationship between digital transformation and food security requires a deeper analysis of the contextual factors that influence this dynamic. Socio-economic disparities play a crucial role in affecting access to technology and its benefits [37]. Marginalized communities often face barriers such as limited internet access, lack of digital literacy, and affordability issues related to technology adoption [14]. These disparities can exacerbate food insecurity by hindering access to information, markets, and resources necessary for efficient food production and distribution. Access to technology also varies by region, and the impact of digital transformation can differ depending on agro-ecological conditions, market structures, and local policies [32]. Addressing these issues requires policies and programs aimed at reducing socio-economic disparities, bridging the digital divide, and adapting digital solutions to the specific context of different regions. Integrating a deeper analysis of these contextual factors enhances our understanding of the relationship between digital transformation and food security, thereby supporting efforts to achieve SDGs [31].

In addition to considering socio-economic disparities, access to technology, and regional variations, it is crucial to discuss the impact of COVID-19 on food security. The COVID-19 pandemic has exacerbated food insecurity globally, disrupting supply chains, increasing prices, and reducing access to nutritious foods, particularly for vulnerable populations [51,52]. Lockdown measures, trade restrictions, and economic downturns have further strained food systems, leading to income loss and reduced food access [53]. Addressing these challenges requires comprehensive responses, including social protection measures, food assistance programs, and efforts to strengthen food systems’ resilience to future shocks through digitalization and local food production initiatives.

The use of digital technologies in agriculture can bring numerous benefits, such as cost reduction, resource optimization, and increased sustainability [54,55,56]. These technologies can be valuable tools for farmers, helping them make better decisions and improving the performance and efficiency of agricultural activities. Moreover, their integration into the agricultural sector can contribute to reducing environmental impact and creating more sustainable and climate-resilient food systems [12]. AI-based systems can provide farmers with valuable information for making better decisions regarding planting, irrigation, and crop management by analyzing meteorological data and soil models. [57]. The use of Big Data in agriculture allows for the collection and analysis of massive amounts of data from various sources, as well as the use of this data to obtain valuable insights for farmers and improve crop and natural resource management [14]. These technologies can contribute to increasing agricultural yields and reducing losses, positively impacting food security and environmental sustainability. These benefits can help strengthen the resilience of the food system, providing solutions and tools to support management and adaptation to future challenges [1,58,59,60]. Farmers can make better-informed decisions and more efficiently adapt agricultural practices to changing environmental conditions and market requirements [37]. Implementing technologies such as artificial intelligence, Big Data, the internet of things, and blockchain in the food system can improve operational efficiency, supply chain transparency, and food safety [6,61,62,63,64,65]. Therefore, exploring and understanding the impact of the digital economy on the food system is an essential step in developing strategies and policies that promote a more robust and adaptable food system to environmental changes.

### 2.2. The Influence of Digitalization on Sustainability in the Food Sector

The digital economy and sustainable development are widely analyzed topics in the literature. There is a growing concern for understanding how technological progress can be strategically directed to support SDGs. Recent studies explore various aspects of this complex interaction, including the impact of digital technologies on the environment, resource efficiency, and social inclusiveness [66,67,68]. Researchers and policymakers aim to find ways to promote sustainable and equitable economic development in the digital age by investigating these aspects. The digital economy provides a new impetus and direction for sustainability [69]. While both sustainable development and the digital economy are significant priorities for governments and international organizations, there are still questions and uncertainties about how these two domains intersect and interact with each other [68].

SDGs proposed by the United Nations reflect a recognition of the need for global action to address significant challenges facing humanity, such as poverty, hunger, inequality, and environmental degradation [70]. All countries and regions of the world must take responsibility and work together to achieve these goals by 2030 to create a more sustainable and prosperous future for all. Integrating sustainable development goals into food systems aims to ensure food and nutritional security for all while protecting the environment and supporting the social and economic well-being of communities. An interdisciplinary and collaborative approach to addressing the complex challenges associated with global food and nutrition is essential for sustainability [68]. Integrating digitalization into the food system can bring significant improvements in achieving SDGs, particularly in terms of eliminating poverty (SDG 1), ensuring food security (SDG 2), improving health and well-being (SDG 3), and reducing inequality (SDG 10). Digital technologies can facilitate access to agricultural information and services, improve food supply chain management, and enhance transparency and food safety. Thus, they can play a crucial role in building a more efficient, equitable, and sustainable food system [13].

SDG 1 Zero Poverty is a fundamental objective of the sustainable development agenda and is crucial in the fight against inequality and social exclusion [71,72]. The use of artificial intelligence can be a valuable tool in poverty alleviation efforts, but it is essential to ensure that these technologies are used fairly and equitably to avoid exacerbating inequality and discrimination [68,73]. A careful and balanced approach is needed to maximize the benefits of digital technologies in supporting SDGs, ensuring that no group or community is left behind in this journey towards a more inclusive and prosperous future [17].

SDG 2 Zero Hunger is a vital goal of sustainable development, and its achievement requires holistic and interdisciplinary approaches to address the complex challenges of the food system [17,74,75]. To combat food insecurity, policies and practices must be adopted to ensure equitable access to food and promote sustainable agricultural practices to ensure long-term food security for all inhabitants of the planet [68,76]. Digitalization and artificial intelligence (AI) are instrumental in mitigating food waste throughout the entire agri-food supply chain, including downstream phases like food consumption. Various tools are employed to measure and manage food waste, facilitating real-time monitoring and analysis [40]. By harnessing the power of digitalization and AI, organizations can enhance inventory management, streamline production processes, and deploy predictive analytics to address and mitigate food waste proactively. Also, Purnhagen et al. [77] indicate that efforts toward achieving SDG2 would benefit from incorporating biotechnology innovations into organic agriculture.

SDG 3 Health and Well-being is essential for improving the quality of life and promoting health [17]. An adequate food system can ensure access to nutritious and nutritionally balanced foods, contributing to preventing malnutrition, nutritional deficiencies, and health problems associated with poor nutrition, such as obesity, cardiovascular diseases, and diabetes. A sustainable food system contributes to protecting the environment and natural resources through responsible agricultural and production practices.

SDG 10 Reducing Inequalities is critical for promoting a fair and inclusive society. Inequality can have profound consequences on well-being and social cohesion, negatively affecting access to opportunities and individuals’ fundamental rights [17]. Economic and social inequality can significantly influence access to healthy and nutritious food, contribute to disparities in health status, and affect food production and agricultural resource distribution [78,79].

In this paper, sustainable development is quantified through the four SDGs, and digital transformation is evaluated through the DESI, a tool used by many researchers [68,69,80]. This investigation focuses on four food-related SDGs—SDG 1, SDG 2, SDG 3, and SDG 10—which concern the resilience and sustainability of the food system from the perspective of the end consumer, simultaneously addressing equal access to food and the health effects of a sustainable food system on individuals. Future research will include other SDGs that address the entire agri-food chain, such as SDG12. This expansion will enable the investigation of aspects concerning responsible production and consumption throughout the food system, including food delivery and packaging. The integration of digitalization with sustainability requires a comprehensive approach that includes concepts from the circular economy and a critical examination of SDG12, focusing on responsible production and consumption. Digital platforms can facilitate collaboration and information sharing among stakeholders, promoting circular economy principles and efficient supply chain management. Integrating digitalization into sustainability initiatives enhances organizations’ ability to achieve SDG12 targets and contribute to broader environmental and social objectives, including mitigating climate change and supporting food security efforts.

The integrated approach, combining the measurement of sustainable development with the evaluation of digital transformation, provides a comprehensive perspective on socio-economic progress and sustainability in the food sector. The first hypothesis of the study concerns the relationship between digitization and food-related SDGs:

**Hypothesis 1.**  *Digital transformation can significantly positively influence the reaching of the food-related SDGs at the EU country level.*

Digital transformation is an emerging paradigm aimed at fundamentally transforming production processes and food processing systems through the integration of advanced digital technologies [38,39,81,82]. This transformation promises to lead to a significant increase in efficiency and product quality and a reduction of losses in the food industry [37,42,83]. However, the approach and level of implementation of digital technologies may vary depending on the context and specific needs of each organization, sector, or country. The influence of the digital economy on sustainable development is multidimensional and cannot be generalized. It is essential to recognize the specific context of each country. Different strategies and policies implemented can influence how the digital economy contributes to sustainable development [13]. Therefore, a more detailed approach and broader analysis are needed to understand this relationship better [17]. The study proposes the second hypothesis based on these considerations:

**Hypothesis 2.**  *European countries can be grouped into homogeneous clusters regarding the digital economy and SDGs related to food.*

## 3. Research Methodology

### 3.1. Selected Data

The data used for investigating the two hypotheses are sourced from reports issued by the European Commission concerning the DESI, as well as SDG reports, and they cover the period from 2017 to 2022 [84,85]. DESI is structured around four primary domains. Each domain reflects fundamental aspects of digitalization within a society. Firstly, Connectivity evaluates a country’s telecommunications infrastructure and its level of digital connectivity, encompassing metrics such as internet speed and broadband and ultra-broadband network coverage, as well as the availability of internet services nationally. Secondly, Digital Public Services assesses the digitization level of public services provided by national and local governments, with a focus on the accessibility and quality of digital services for citizens and businesses, spanning areas such as e-governance, e-health, and e-education. Thirdly, Human Capital refers to the digital skills level of the population and the requisite human capabilities for effective utilization of digital technologies, encompassing access to digital education and training, as well as the extent of digital skill utilization in everyday life and the workplace. Lastly, Digital Technology Integration examines the degree of digitalization within businesses and the private sector overall, as well as the adoption of digital solutions across various economic sectors. DESI is calculated by the European Commission using a specific methodology, which involves assessing a set of relevant indicators for digitalization. The calculation methodology may vary depending on the specific components included in the index and the weights assigned to them. Generally, DESI calculations may involve a combination of statistical data, reports, and indicators collected from the EU member states, as well as other relevant sources. The final calculation of the DESI is the result of aggregating all these components and indicators, using either complex mathematical formulas or other weighting and aggregation methods [85].

The SDG report is an assessment document that tracks the progress and performance of countries towards achieving the SDGs set forth in the 2030 Agenda for Sustainable Development. These reports are typically compiled and published by various international organizations, including the United Nations and its agencies, as well as other intergovernmental and non-governmental organizations. They offer an overview of each country’s performance in the context of its commitment to sustainable development and aim to identify achievements and obstacles encountered. The indices for measuring the SDGs are calculated based on a set of indicators relevant to each goal. These indicators are selected to capture various aspects of sustainable development, such as poverty, health, education, gender equality, climate change, and environmental protection. The calculation of SDG indices involves aggregating data from national statistics offices, international databases, and other sources to assess the progress of countries towards achieving each goal. The indices are typically presented as scores or rankings, allowing for comparisons between countries and tracking progress over time [84].

The study focused on four goals related to the food system: SDG1, SDG2, SDG3, and SDG10. Table 1 presents the research variables, measures, period, and data collection sources.

### 3.2. Methods

The datasets retrieved from the SDG report [84] and the European Commission [85] spanning the timeframe of 2017–2022 were aggregated into a centralized database serving as the fundamental framework for subsequent statistical analyses. Investigation of the two hypotheses regarding the influence of digitalization on sustainability in the food system involves two methods: structural equation modeling and cluster analysis. For investigating the Hypothesis H1, structural equation modeling is the appropriate method due to its ability to examine the complex relationships among multiple variables and to evaluate the direct and indirect impact of factors on the final variables. SEM allows for the modeling of latent variables, which are unobserved constructs inferred from multiple observed variables. These latent variables represent underlying concepts or constructs that cannot be directly measured. According to Hair et al. [86] and Garson [87], SEM is ideal for testing and validating complex theoretical models involving multidimensional interactions between observed and unobserved variables. Using these constructs, SEM enables the examination of causal relationships among variables, allowing researchers to assess the direct and indirect effects of one variable on another within a hypothesized model. SEM enables the examination of the impact of different components of digitalization on food security objectives and food-related SDGs at the EU country level. This approach allowed us to assess the complexity and interdependence among the variables involved in the relationship between digital transformation and sustainability in the food system [86,87].

The model developed to quantify the relationships between the components of DESI, SDGs related to foods, and the overall SDG index is formative. The latent variables of the model are the DESI, SDG Index, and SDGs related to food. Formative SEM models are a variant of SEM that treats endogenous variables as being composed of observable variables contributing to the construction of the latent concept. In this type of model, endogenous variables are considered to be constructed directly based on exogenous variables [87]. In the second phase, the model was modified to determine the individual influences of the digital economy on SDGs related to food, defining as latent variables GOAL 1: No Poverty, GOAL 2: Zero Hunger, GOAL 3: Good Health and Well-being, and GOAL 10: Reduced Inequality, alongside the SDG Index and Digital Economy and Society Index.

Investigating the Hypothesis H2 required the use of cluster analysis [88]. Cluster analysis, a statistical technique employed in this study, aims to identify clusters within a dataset, in this case, to explore the influence of digitalization on sustainability in the food system among European countries. This method involves selecting relevant variables, applying clustering algorithms to partition the dataset into distinct groups, and interpreting the resulting clusters to discern underlying patterns or similarities among countries. Cluster analysis enables the investigation of whether there are common patterns or trends among EU countries regarding the level of digitalization and their performance in achieving food-related SDGs. This approach helped us better understand the diversity and similarities among EU member states concerning digital transformation and their progress in promoting sustainable and equitable food systems.

## 4. Results

**Figure 1:**
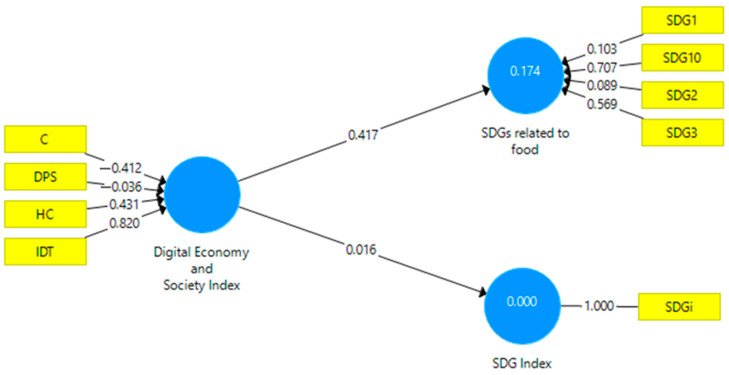
Empirical model. Source: Author’s design based on data using SmartPLS v.3.0.

Hypothesis H1 used structural equation modeling in the partial least squares variant. The software used is SmartPLS v3.0 [89]. Figure 1 presents the empirical model created to quantify the relationships between the components of DESI, SDGs related to food, and the overall SDG index. The relationship between these components is formative, indicating that they collectively contribute to the overall assessment of SDGs related to food.

An essential indicator of the validity and reliability of a formative SEM model is the variance inflation factor (VIF). VIF is a measure used to assess the degree of collinearity among explanatory variables. Collinearity is a problem when two or more independent variables in a regression model are strongly correlated with each other. This correlation can lead to misunderstandings in interpreting path coefficients and their precision [86]. Table 2 presents the VIF values for the SEM model.

Table 2 reveals that the VIF values are below 3, indicating good model validity. SRMR (standardized root mean square residual) and NFI (normed fit index) are measures used in SEM model analysis to assess how well the model fits the observed data and to make adjustments and improvements based on the specific research needs and available data [87]. In the proposed SEM model, SRMR has a value below 0.08 (0.042), and NFI has a value above 0.9 (0.906). The model is adequate and fits well with the observed data.

A bootstrap procedure with bias-corrected, bidirectional, and significance level set at 0.05 enables the calculation of path coefficients in order to test Hypothesis H1. Bootstrap is a technique of repeated sampling of observations from the dataset with replacement, allowing for estimation of the sampling distribution of a statistic [86]. This method generates robust estimates of path coefficients and their associated standard errors, which are essential for assessing the significance of relationships between variables. Table 3 presents the path coefficients of the SEM model.

The use of a bidirectional approach allows for the examination of both positive and negative effects, capturing the entire spectrum of possible relationships between digital transformation and SDGs related to food. Table 3 presents the path coefficients for the relationships between DESI, the SDG Index, and the food-related SDGs. The path coefficient for the relationship between DESI and the SDG Index is 0.016. This coefficient indicates a weak association between the Digital Economy and Society Index and the SDG Index, and the high *p*-value (0.889) suggests that this association is not statistically significant.

In contrast, the path coefficient for the relationship between DESI and the food-related SDGs is 0.417. This value indicates a significant and positive association between the Digital Economy and Society Index and the food-related SDGs. Furthermore, the *p*-value < 0.001 suggests that this association is statistically significant. The results show that digitalization, illustrated by DESI, has a significant and positive impact on achieving food-related SDGs, although the association with the overall SDG Index is not statistically significant.

**Figure 2:**
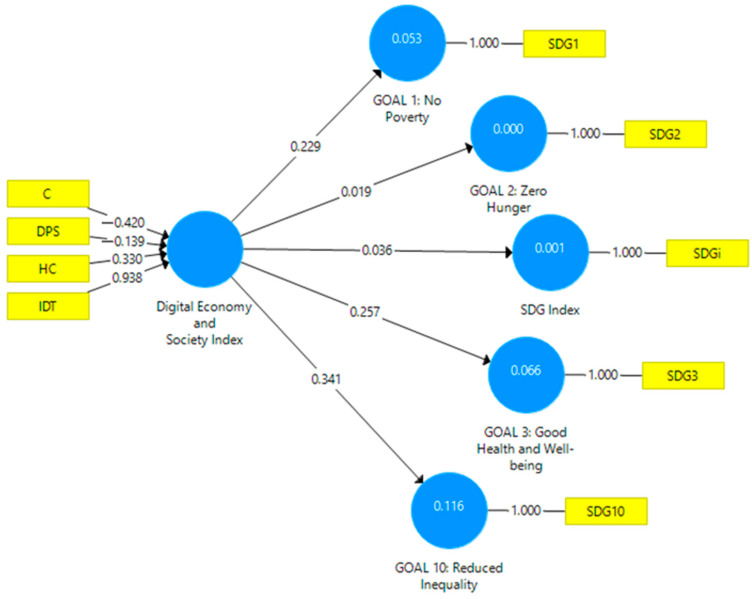
Empirical model modified. Source: Author’s design based on data using SmartPLS v.3.0.

The model was modified to determine the individual influences of the digital economy on SDGs related to food, defining latent variables such as GOAL 1: No Poverty, GOAL 2: Zero Hunger, GOAL 3: Good Health and Well-being, GOAL 10: Reduced Inequality, alongside the SDG Index and Digital Economy and Society Index. The SEM model remains formative (Figure 2).

Also, in this model, VIF has values below 3, indicating the model’s good validity (Table 4).

In the modified SEM model, SRMR has a value below 0.08 (0.04), and NFI has a value above 0.9 (0.913). The model is adequate and fits the observed data well.

The bootstrap procedure, being bias-corrected, bidirectional, and having a significance level of 0.05, generated the path coefficients of the modified SEM model. Table 5 presents the path coefficients of the modified SEM model.

The analysis of path coefficients shows mixed results regarding the significance of the individual influences of the digital economy on achieving SDGs related to food. For SDG 10, the path coefficient is 0.341, with a standard deviation of 0.108. The T-value is 3.165, indicating statistical significance at a confidence level of 0.002, underscoring a significant relationship between the Digital Economy and Society Index and the goal of reducing inequality. In the case of SDG 1, the path coefficient is 0.229, with a standard deviation of 0.11. The T-value is 2.085, with a *p*-value of 0.038, indicating a significant relationship between the DESI and the goal of poverty eradication, but at a lower level of significance. Investigating the relationship between DESI and SDG 2 reveals that the path coefficient is 0.019, with a standard deviation of 0.14. The T-value is 0.134, and the *p*-value is 0.893, indicating no significant relationship between the DESI and the goal of hunger alleviation. The influence of DESI on SDG 3 shows that the path coefficient is 0.257, with a standard deviation of 0.102. The T-value is 2.524, with a *p*-value of 0.012, indicating a significant relationship between DESI and the goal of promoting health and well-being. The influence of DESI on the SDG Index generates a path coefficient of 0.036, with a standard deviation of 0.104. The T-value is 0.347, and the *p*-value is 0.729, indicating that, as in the case of the initial model, there is no significant relationship between the DESI and the overall SDG Index. These results suggest that digital transformation can have a significant impact on specific SDGs, such as poverty alleviation, reducing inequality, and improving health and well-being. However, it may not significantly influence hunger alleviation.

The results of the investigations conducted using SEM models partially validate Hypothesis H1. DESI influences the reach of the food-related SDGs overall, but individual influences are significant in the cases of SDG 1, SDG 3, and SDG 10. In the case of SDG 2, no significant influences were found. These findings suggest that the adoption and development of digital technologies could play an essential role in poverty reduction and improving the health and well-being of the population. However, concerning hunger alleviation, the impact of digital technologies may be more limited or influenced by other variables and specific contexts. It is essential to continue exploring and investigating the role and potential of digital technologies in achieving SDGs and to identify effective ways to integrate them into the food system to promote a more equitable, sustainable, and resilient approach.

**Figure 3:**
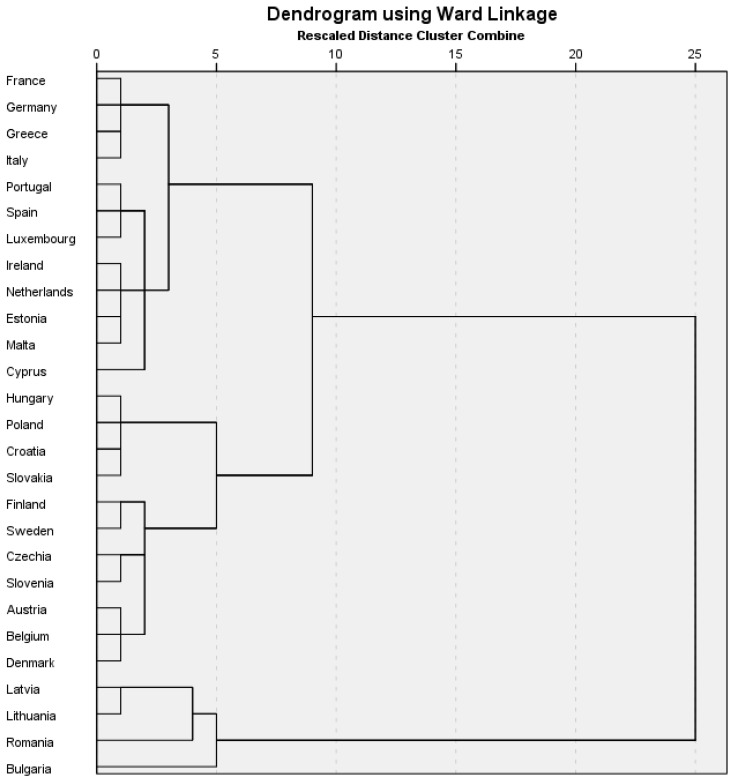
Dendrogram. Source: Author’s design based on data using SmartPLS v.3.0.

Hypothesis H2 involves cluster analysis conducted within EU countries based on the digital economy illustrated by DESI and sustainability in the food domain illustrated by SDGs related to food. Ward’s method, which used squared Euclidean distance intervals, was an adequate choice for cluster analysis. Figure 3 illustrates the dendrogram depicting the three clusters obtained. Ward’s method is known for its ability to form compact and homogeneous groups that are easy to interpret [90]. This method minimizes the variation within each cluster and maximizes the variation between clusters, leading to the formation of well-defined and distinctive groups. Using squared Euclidean distance is suitable for continuous data and can efficiently measure differences between observations in multidimensional space.

Table 6 presents the three distinct clusters, labeled A, B, and C, built using Ward’s method, which include countries grouped homogeneously based on indicators of the digital economy and sustainability in the food area. When comparing these clusters with the EU mean, we can observe the differences and similarities among the member countries of the European Union regarding the level of digitization and progress in achieving SDGs.

All countries in the three clusters stand out with high values for SDG1, close to the EU average, illustrating a good situation for EU countries in combating poverty.

Cluster A comprises countries characterized by relatively high values of connectivity, digital public services, human capital, and digital technology integration indicators. These countries, such as France, Germany, and Spain, stand out for their efforts in combating poverty (SDG1) and ensuring good health and promoting well-being (SDG3). However, most of these countries are below the European mean in reducing inequality (SDG10), and half of the countries in cluster A fall below the EU average in achieving zero hunger (SDG2).

In Cluster B, values are more moderate but still significant, suggesting a medium level of digital technology development and progress in achieving SDGs. These countries, such as Belgium, Austria, and Finland, demonstrate a solid commitment to achieving food-related SDGs as well as advancing their digital capabilities. The cluster’s average for all SDGs related to food is around or above the EU average. On the other hand, Cluster C includes countries with lower values of digitalization indicators and modest achievements in SDGs. These countries, such as Romania and Bulgaria, face significant challenges, particularly in promoting health and well-being and countering inequality.

The results of the cluster analysis indicate that European countries can be grouped into homogeneous clusters regarding the digital economy and food-related SDGs, thus validating Hypothesis H2.

## 5. Discussion

A variety of factors influence the food system, and its resilience depends on its ability to cope with these influences and respond efficiently to disruptions [91,92]. Identifying and understanding these factors is essential for developing and implementing strategies to support a more resilient and adaptable food system to changes and crises that may arise [93,94]. Evaluating and addressing these factors allows academics to contribute to building a safer and more sustainable food system for the future [6,95,96].

Agriculture and the food industry play an essential role in the global and regional economy, driving development and employment in many countries [97,98]. However, these sectors face significant challenges in adapting to new technologies and trends, as well as understanding how digital transformation can improve the efficiency and sustainability of the entire food chain [99,100].

The integration of digital technologies in the food domain not only improves the efficiency and quality of production and distribution processes [59] but also contributes to increasing the resilience of the food system by facilitating adaptation to changes in the business environment and market requirements [12]. The use of digital technology can radically transform the way food operations are managed, allowing for greater flexibility and efficiency in the face of continuously changing challenges and opportunities [101].

According to this research results, digitalization illustrated by DESI has an impact on achieving food-related SDGs, but this impact is more significant in some areas than others, partially validating Hypothesis H1. DESI significantly and positively influences goals aimed at poverty eradication, health promotion, and inequality reduction (SDG1, SDG3, and SDG10). However, for SDG 2, which aims to combat hunger, no significant influences of DESI were observed. This finding highlights that the impact of the digital economy on various issues of sustainable development in the food domain can vary and sometimes even be contradictory, as shown by Wang et al. [6], Bachmann et al. [17], Baierle et al. [32], and Kuhn [78]. DESI significantly and positively influences SDGs aimed at poverty eradication and health promotion, findings that support those of Bachmann et al. [17]. These results suggest that the adoption and development of digital technologies could contribute to poverty reduction and improve the health and well-being of the population [68]. However, no significant influences were found regarding SDG2. This result may indicate that the impact of digital technologies on addressing hunger issues may be limited or influenced by other specific factors and contexts [32,36].

The integration of advanced technologies into production and distribution processes brings significant benefits, improving the efficiency, quality, and sustainability of the entire food production chain [102]. Furthermore, bringing innovation and digital transformation into the food sector can contribute to economic growth and sustainable development of communities, offering opportunities for farmers, producers, and consumers alike [103]. However, it is essential to recognize that the adoption process of digital technologies may differ depending on the economic, social, and cultural context of each country [26]. Therefore, it is essential to understand the particularities and specific needs of each environment where these technologies are implemented to ensure an efficient and fair transition to a more innovative and sustainable agriculture and food industry [32].

The results of the cluster analysis show a significant correspondence between the performance of countries regarding the digital economy and their progress in achieving SDGs related to food, validating Hypothesis H2 in line with the findings of previous research [6,12,13,59]. This finding emphasizes the importance of adopting and implementing digital technologies to promote a more sustainable agriculture and food industry and contribute to achieving SDGs globally.

Despite global expansion, IT infrastructures are still inadequate in many regions, particularly in developing countries [17]. The rise in inequality affects emerging and developing countries because they do not benefit from the implementation of digital technologies. The high upfront costs associated with implementing robotic systems can pose a barrier to entry, particularly for small and medium-sized family farms. This discrepancy in access to advanced technologies could widen the gap between larger, more financially secure farms and smaller, resource-constrained operations. This digital divide can aggravate global divisions and hinder equal access to the benefits of technology. By acknowledging these disparities, policymakers and stakeholders can work towards devising strategies to ensure equitable access to technology and mitigate the risk of widening inequality within the agricultural sector. The results of the cluster analysis conducted among EU countries, including cluster C (such as Romania and Bulgaria), revealed a negative influence of low levels of digitization on high levels of inequality. A balanced and collaborative approach is necessary to address these issues and promote a more equitable and sustainable technological development globally [32].

Therefore, it is evident that the advancement of digital technology is a critical factor in strengthening the resilience of the food system and promoting a more sustainable and adaptable agriculture to changes in the surrounding environment and market demands [104,105,106]. Governments, the private sector, and other stakeholders must continue to invest in the development and implementation of digital technologies in agriculture and food to ensure a safer and more prosperous future for all involved in the food chain [107].

### 5.1. Theoretical Implications

Improving and transforming food processing systems are essential to meet increasing demand and achieve sustainability and resilience goals in the food industry. Deep collaboration among industry stakeholders, researchers, and government entities is imperative for the advancement and adoption of cutting-edge technologies and methodologies in food processing. Innovations like automation and connectivity play pivotal roles in tackling the escalating challenges within the food sector, offering ways of creating heightened efficiency, accuracy, and sustainability in food processing operations. These technological breakthroughs hold the potential to bolster food safety measures, elevate product standards, and diminish food loss and waste, thereby facilitating a more resilient and resource-efficient food industry.

The integration of digital technologies into the agricultural sector brings multiple benefits, ranging from improving operational efficiency and increasing productivity to reducing negative environmental impact and promoting more sustainable agriculture. These technologies can help farmers manage resources more efficiently, optimize production processes, and reduce dependence on external inputs, thereby contributing to more efficient use of natural resources. Implementing digital technologies can improve access to information and services in rural areas, thereby reducing disparities and promoting equitable development in the agricultural sector.

The research results underscore the importance of continuing research efforts and implementing digital technologies in the food sector, with particular attention to how these technologies can contribute to achieving SDGs. It is crucial to continue investigating and exploring how digital technologies can be integrated and efficiently utilized to address the complex challenges related to food and promote a fairer, more sustainable, and more resilient food system.

### 5.2. Empirical Implications

The evolution of digital technology in the food sector brings significant benefits not only in terms of efficiency and productivity but also in enhancing the adaptability and resilience of the entire food system. Digital technologies enable process optimization and waste reduction, thereby enhancing the sustainability and efficiency of the entire food supply chain. This study underscores the significance of precisely evaluating the digital economy’s impact on the sustainable evolution of the food system. By examining the influence of the digital economy illustrated by DESI on key food-related SDGs, namely SDG 1, SDG 2, SDG 3, and SDG 10, the research highlights a clear correlation between the advancement of the digital economy and the strides made toward achieving these crucial sustainable development objectives.

The research results show that DESI has mixed effects on food-related SDGs (SDG1, SDG2, SDG3, and SDG10). While significant influences of DESI are observed on SDG1, SDG3, and SDG10, it seems to have a smaller or nonsignificant impact on SDG2. It is essential to understand that digital transformation, although it may have a positive impact on specific aspects of SDGs, is not a panacea and cannot solve all issues related to sustainable development in the food domain. Thus, countries’ governments need to be aware of this complexity and adopt well-adjusted, multidimensional approaches to endorse sustainable development. Furthermore, these results suggest that further analysis is needed to understand better how the digital economy can contribute to achieving food-related SDGs.

Understanding how digital technologies can contribute to achieving these objectives enables policymakers to develop and implement more effective strategies (policy interventions, investments in digital infrastructure, and capacity-building initiatives) to address complex challenges related to food security, poverty, nutrition, and inequality. Policy interventions encompass regulatory measures and governmental actions aimed at addressing specific issues or achieving particular goals. In the context of enhancing food security through digital transformation, policy interventions might involve implementing regulations to ensure equitable access to digital technologies, incentivizing the development and adoption of digital tools for agriculture and food distribution, and establishing frameworks for data governance and privacy protection.

Investments in digital infrastructure refer to financial commitments made by governments, private sector entities, or international organizations to improve the technological foundation necessary for digital transformation. These investments could include funding for expanding broadband internet access to rural and underserved areas, establishing digital platforms for farmers to access market information and financial services, and supporting research and development in agri-tech innovations.

Capacity-building initiatives involve activities aimed at enhancing the knowledge, skills, and capabilities of individuals, organizations, and communities to effectively utilize digital technologies for improving food security outcomes. These initiatives may include training programs for farmers and agricultural extension workers on how to use digital tools for crop management, supply chain optimization, and market access. Additionally, capacity-building efforts could focus on building the technical expertise of government agencies and non-governmental organizations to design and implement digital solutions that address the unique needs and challenges of different regions and communities.

### 5.3. Limitations and Further Research

Although this research makes significant contributions to understanding the relationship between digital transformation and food security, there are several limitations to consider. This research is based on DESI to measure digital transformation. However, other dimensions of digital transformation could be included in the analysis to obtain a more comprehensive picture. Also, generalizing the results would require further investigation outside the European Union to confirm and validate the findings. Alternative research methodologies could also be examined to complement and validate the results obtained in this study. Using more up-to-date and comprehensive data could strengthen the robustness of the analysis.

Concerning further research, contextual analysis of the influence of socio-economic and cultural factors, evaluating the impact of policy interventions, and extending the analysis could provide a more comprehensive and detailed perspective on this complex relationship. Using diverse research methods, such as mixed approaches or case studies, could also contribute to a deeper understanding of the phenomenon and provide richer data and information.

In addition to addressing the four food-related SDGs (SDG1, SDG2, SDG3, and SDG10), integrating insights from other SDGs, such as SDG12, focusing on sustainable consumption and production patterns, is crucial. Promoting responsible production practices, including waste reduction and minimizing environmental impact, empowers the agri-food sector to significantly contribute to achieving SDG12 targets by implementing efficient production methods and adopting eco-friendly technologies. Addressing food waste is paramount for mitigating hunger, enhancing food security, and ensuring food safety, requiring measures such as improving infrastructure, implementing better inventory management, and raising consumer awareness about waste reduction. Integrating responsible production and consumption practices, alongside efforts to reduce food waste, is essential for achieving SDGs related to food security, environmental sustainability, and social equity, enabling the agri-food sector to play a pivotal role in building a more resilient and sustainable food system for the future.

## 6. Conclusions

In the contemporary era marked by rapid technological advancement, the significance of digital transformation in bolstering food security and advancing SDGs is of vital importance. This paper underscores the pivotal role of digital transformation in bolstering food security and propelling SDGs forward. The integration of digital technologies into the food sector stands as pivotal for enhancing efficiency, productivity, and sustainability throughout the entire food chain. Technological innovations such as artificial intelligence, robots, remote sensors, connectivity, and internet use wield the potential to optimize processes, curtail food loss and waste, and foster more sustainable agricultural practices.

A thorough evaluation of how the digital economy influences the sustainable development of the food system provides the foundation for creating appropriate policies and strategies. Unraveling how various facets of the digital economy intersect with specific dimensions of sustainable development yields invaluable insights for grappling with the intricate challenges in the realm of food. Policymakers and decision-makers must heed the findings of such research endeavors to drive sustainable and resilient development within the food sector, not only in the European Union but also across the globe.

Digital transformation presents substantial opportunities for bolstering food security and advancing SDGs. Capitalizing on these opportunities enables the effort to strive toward constructing a more efficient, equitable, and sustainable food system. This paper contributes to the scientific discourse by highlighting the pivotal role of digital technologies in reshaping the future of food, thereby preparing the ground for innovative strategies and interventions aimed at addressing pressing global challenges.

## Figures and Tables

**Table 1:** Research variables, datasets, measures, timeframe, and sources.

Variable	Dataset	Measures	Timeframe	Sources
C	Connectivity	Weighted score (0 to 100)	2017–2022	[85]
DPS	Digital Public Services	Weighted score (0 to 100)	2017–2022	[85]
HC	Human Capital	Weighted score (0 to 100)	2017–2022	[85]
IDT	Integration of Digital Technology	Weighted score (0 to 100)	2017–2022	[85]
SDG1	GOAL 1: No Poverty	Weighted score (0 to 100)	2017–2022	[84]
SDG2	GOAL 2: Zero Hunger	Weighted score (0 to 100)	2017–2022	[84]
SDG3	GOAL 3: Good Health and Well-being	Weighted score (0 to 100)	2017–2022	[84]
SDG10	GOAL 10: Reduced Inequality	Weighted score (0 to 100)	2017–2022	[84]
SDGi	SDG Index Score	Weighted score (0 to 100)	2017–2022	[84]

Source: Developed by the author based on [59,60,61,62,63,64,65,66,67].

**Table 2:** Variance Inflation Factor.

	VIF
C	1.316
DPS	1.363
HC	1.382
IDT	1.585
SDG1	1.306
SDG2	1.006
SDG3	1.017
SDG10	1.302
SDGi	1.000

Source: Author’s design based on data using SmartPLS v3.0.

**Table 3:** Path coefficients.

	Original Sample	Sample Mean	Standard Deviation	T Statistics	*p* Values
Digital Economy and Society Index- > SDG Index	0.016	0.009	0.117	0.140	0.889
Digital Economy and Society Index- > SDGs related to food	0.417	0.47	0.069	6.063	0

Source: Author’s design based on data using SmartPLS v.3.0.

**Table 4:** Variance Inflation Factor of modified model.

	VIF
C	1.316
DPS	1.363
HC	1.382
IDT	1.585
SDG1	1.306
SDG2	1.006
SDG3	1.017
SDG10	1.302
SDGi	1.000

Source: Author’s design based on data using SmartPLS v3.0.

**Table 5:** Path coefficients of modified model.

	Original Sample	Sample Mean	Standard Deviation	T Statistics	*p* Values
Digital Economy and Society Index → GOAL 1: No Poverty	0.229	0.23	0.11	2.085	0.038
Digital Economy and Society Index → GOAL 2: Zero Hunger	0.019	0.022	0.14	0.134	0.893
Digital Economy and Society Index → GOAL 3: Good Health and Well-being	0.257	0.273	0.102	2.524	0.012
Digital Economy and Society Index → GOAL 10: Reduced Inequality	0.341	0.342	0.108	3.165	0.002
Digital Economy and Society Index → SDG Index	0.036	0.023	0.104	0.347	0.729

Source: Author’s design based on data using SmartPLS v.3.0.

**Table 6:** Hierarchical clusters.

	C	DPS	HC	IDT	SDG1	SDG2	SDG3	SDG10
France	16.05	16.84	12.47	7.98	99.7	72.4	93.2	87.5
Germany	16.83	15.85	11.24	8.96	99.5	72.4	93.0	88.1
Greece	12.39	9.85	10.03	6.66	99.2	66.6	90.3	84.6
Italy	15.31	14.62	9.14	10.19	97.5	69.8	93.9	77.9
Portugal	12.90	16.98	11.49	9.40	99.9	64.3	92.1	84.4
Spain	17.43	20.88	12.83	9.63	98.7	65.4	94.2	81.4
Luxembourg	14.83	20.84	14.44	8.74	100.0	58.9	96.5	84.0
Ireland	15.38	20.86	15.66	10.83	99.9	67.7	94.4	90.3
Netherlands	17.53	21.05	15.78	13.02	99.3	67.7	95.7	89.8
Estonia	11.11	22.79	13.49	9.12	100.0	63.2	89.5	89.1
Malta	13.25	21.45	14.15	12.03	99.8	66.3	91.2	86.6
Cyprus	14.69	14.38	10.44	8.84	99.9	53.7	91.1	85.5
Cluster A mean	14.81	18.03	12.60	9.62	99.46	65.68	92.92	85.77
Hungary	14.40	14.35	9.61	5.40	98.9	70.3	83.6	92.7
Poland	11.63	13.94	9.26	5.72	99.0	67.5	85.2	93.4
Croatia	12.01	13.39	12.96	9.18	100.0	74.3	86.4	94.2
Slovakia	12.46	13.00	11.03	6.96	99.2	72.3	87.8	100.0
Finland	15.14	21.84	17.85	14.77	99.6	60.9	95.4	98.5
Sweden	15.06	20.61	15.49	14.06	98.9	63.1	96.9	95.0
Czechia	13.17	16.11	11.40	8.46	99.9	62.1	90.2	100.0
Slovenia	14.97	17.37	11.06	9.96	99.4	66.6	92.4	100.0
Austria	14.12	18.03	12.74	9.79	99.5	73.1	92.5	94.6
Belgium	9.96	16.19	12.17	11.99	99.5	71.2	93.4	100.0
Denmark	19.27	20.77	14.80	14.50	99.2	71.0	95.4	98.2
Cluster B mean	13.84	16.87	12.58	10.07	99.36	68.40	90.82	96.96
Latvia	12.52	19.70	11.03	6.46	100.0	64.2	84.3	72.6
Lithuania	12.34	20.45	10.61	9.31	100.0	59.6	86.1	70.9
Romania	13.81	5.26	7.73	3.79	98.6	72.9	80.6	77.2
Bulgaria	12.68	12.97	8.15	3.88	100.0	68.2	79.3	51.0
Cluster C mean	12.83	14.60	9.38	5.86	99.63	66.25	82.56	67.95
EU mean	14.12	17.05	12.11	9.24	99.45	66.87	90.53	87.69

Source: Authors’ design based on data using SPSS v.27.

## Data Availability

Research data are publicly available: https://digital-strategy.ec.europa.eu/en/library/digital-economy-and-society-index-desi-2022, accessed on 12 February 2024; https://dashboards.sdgindex.org/static/downloads/files/SDR2023-data.xlsx, accessed on 12 February 2024.
